# Leading through performance crises: soccer coaches’ insights on their strategies—a qualitative study

**DOI:** 10.3389/fpsyg.2025.1576717

**Published:** 2025-04-02

**Authors:** Constantin Rausch, Julian Fritsch, Stefan Altmann, Lena Steindorf, Jan Spielmann, Darko Jekauc

**Affiliations:** ^1^Department of Health Education and Sport Psychology, Institute of Sports and Sports Science, Karlsruhe Institute of Technology, Karlsruhe, Germany; ^2^Department of Sports Psychology, Institute of Sports Sciences, Goethe University, Frankfurt, Germany; ^3^TSG ResearchLab gGmbH, Zuzenhausen, Germany

**Keywords:** soccer, leadership, crises, management, coach, qualitative study

## Abstract

**Introduction:**

Performance crises in sports are recognized as particularly stressful environments, where coaches are held responsible to a large extent for winning matches. During these challenging times, coaches play a crucial role, as their behaviors can significantly impact the course of a crisis, either improving or exacerbating the situation. Therefore, the aim of the current study was to explore the various roles professional soccer coaches adopt during a performance crisis in order to manage them.

**Methods:**

Sixteen semi-structured interviews were conducted with professional soccer coaches aged between 32 to 54 years (*M* = 43.81, SD = 6.46), with coaching experience ranging from 7 to 23 years (*M* = 15.44, SD = 5.0). The qualifications of the participants included eleven UEFA Pro Licenses, two UEFA A Licenses, one UEFA Goalkeeping A License, and two coaches without a UEFA License. Using an inductive reflexive thematic analysis within a broader deductive framework, two fundamental roles (i.e., Self-Manager, People-Manager) and three soccer-specific roles (i.e., the Soccer Expert, the Psychologist, the Administrator) were identified.

**Results:**

The roles of the Self-Manager and People-Manager are essential for coaches to understand, regulate, and influence themselves and others, enabling them to effectively perform the specific behaviors associated with each soccer-specific role. The Soccer Expert encompasses soccer-specific knowledge and expertise, the Psychologist focuses on addressing the psychological needs of both individual players and the entire team, and the Administrator is characterized by overseeing the administrative and organizational elements.

**Discussion:**

Emphasizing the interpersonal dimension of coaching, advanced self-management and communication competencies are particularly highlighted. Overall, by exploring coaches’ experiences, this study may contribute to the growing body of literature on crisis management in sports and offers practical implications for coaches to support themselves and their players during performance crises.

## 1 Introduction

Soccer is one of the most popular sports in the world. The popularity of soccer comes with high expectations and pressure for the individuals involved. Benz and Gehring (2009) depict the professional soccer world as a cycle of never-ending coach dismissals, associated with a high degree of job insecurity and a lack of sustainability at many clubs. As an extreme example, Claudio Ranieri, awarded coach of the year for leading Leicester City F.C. to the championship in the 2015/16 Premier League season, was dismissed 8 months after the sensational championship due to a period of poor results. This example illustrates that coaches operate in a highly volatile environment, as high-performance coaches are held responsible by various stakeholders such as fans, media, and/or club management for achieving poor results (Nash and Mallett, 2019). This accountability for achieving poor results could explain the high coach dismissal rate in professional soccer (Sousa et al., 2024a). For example, Gómez et al. (2021) reported that during the 2017/18 season alone, 55 coaches were dismissed within the “Big Five” European soccer leagues, while Tozetto et al. (2019) found that 87 out of 120 head coaches were dismissed in the top Brazilian soccer league between 2012 and 2017. This high frequency of dismissals is problematic as it significantly impacts not only the coaches but also the players. The constant changes require players to mentally, physically, and technically-tactically adapt to new environments, which, due to emotional and psychological exhaustion, can negatively affect their performance and well-being (Sousa et al., 2024b).

Mid-season coach dismissals are often a consequence of performance crises in sports (Jekauc et al., 2024; Sousa et al., 2024a), which are defined as a “continuous underperformance across multiple games, accompanied by team members’ threat states and the inability of a team to effectively cope with this threat, resulting in low team functioning” (Buenemann et al., 2023, p. 4). Applied to professional soccer, Jekauc et al. (2024) identified that performance crises typically originate from deviations from expected and actual outcomes, often triggered by negative results. These deviations elicit negative affective states, which, in turn, activate a series of psychological processes at both the individual and team level. According to the authors, the psychological processes serve as the sustaining factor of performance crises, as they negatively impact the on-field behavior (e.g., defensive mindset), thereby reducing the likelihood of improved performance. This dynamic creates a self-reinforcing cycle, where poor results further exacerbate psychological distress, ultimately intensifying the crisis. Furthermore, the authors stated that coaches have a major role in managing such crises as their behavior can either improve or worsen the situation. Specifically, it was reported that coaches need to balance external factors (e.g., unrealistic media expectations), team processes (e.g., conflicts within a team), and intraindividual processes (e.g., decreased self-confidence). Considering these various challenging tasks, the focus of the present article is on describing the different roles, reflecting the self-described performance expectations of their responsibilities, that coaches adopt to manage a drop in the team’s performance.

An important construct related to the coaches’ ability to deal with performance issues of their athletes is coaching effectiveness defined as “the consistent application of integrated professional, interpersonal, and intrapersonal knowledge to improve athletes’ competence, confidence, connection, and character in specific coaching contexts” (Côté and Gilbert, 2009, p. 316). Professional knowledge refers to sport-specific aspects, such as technical and tactical skills. Interpersonal knowledge facilitates effective interactions within groups and among individuals, enhancing communication with various stakeholders, while intrapersonal knowledge reflects the ability to review, revisit, and reflect on one’s own actions. Although coaches’ knowledge and the resulting athletes’ outcomes (i.e., competence, confidence, connection, and character) are central to the integrative conceptualization of coaching effectiveness, the specific context shapes these elements.

The coaching context encompasses unique needs and demands, characterized by the performance level and developmental issues within which coaches operate, defining it as either participation-focused by promoting positive sporting experience and involvement (recreational, developmental) or performance-focused by promoting performance outcomes (elite; Cruickshank and Collins, 2015; Lyle, 2002). However, it is important to note that coaches can integrate elements of both approaches simultaneously. For example, while focusing primarily on performance outcomes, a coach can still promote positive sport experiences to ensure athlete well-being and development. Therefore, researchers stress the importance of defining the uniqueness of the context as it has a major impact on the coaching roles and behaviors due to distinct and variable environmental demands (Horton, 2014; Lyle, 2002; Vella et al., 2010). The diversity of tasks coaches have to manage is also reflected in the conceptualization of the term ‘role’ by Lyle (2002). Specifically, roles describe the spectrum of actions and approaches informed by the coach’s comprehension of their responsibilities, tailored to specific circumstances, all aimed at increasing or maintaining performance levels of their athletes.

Elite coaches who operate in a primarily performance-focused context are influenced by various organizational, contextual, interpersonal, and intrapersonal stressors related to their own performance and the performance of their players (Olusoga et al., 2009; Thelwell et al., 2008). In this chaotic and unpredictable context, effective coaching is highly complex (Nash and Mallett, 2019). The constant pressure from various internal and external factors, combined with the need to achieve positive results, highlights the need for coaches to be strategic in their actions, requiring them to navigate a spectrum of different roles and behaviors. Previous research stated that coaches have to take on many different roles in their position, which include that of a performer, educator, administrator, leader, planner, motivator, negotiator, manager, listener, mentor, role model, friend, community leader, etc. (e.g., Bloom et al., 2014). However, considering the specific demands of each coaching context, it becomes clear that such a generic role description must be placed in its specific context in order to effectively capture the diverse environmental demands coaches have to manage. A context that seems particularly relevant for analyzing the diverse environmental demands from an applied perspective is performance crises (Jekauc et al., 2024) as it can be regarded as a severe form of a stressor (Buenemann et al., 2023). If not effectively managed by coaches, the various demands and internal and external stressors during a performance crisis could adversely impact both their own performance and that of their athletes (Buenemann et al., 2023; Cook et al., 2021). Thus, considering the need to examine coaching behaviors in a specific context (Mallett and Rynne, 2015), the purpose of the present study was to explore the different roles coaches adopt during a performance crisis in professional soccer in order to manage the crisis.

## 2 Materials and methods

### 2.1 Philosophical perspectives and design

A qualitative research design was used to explore coaches’ perspectives on their roles and behaviors during a performance crisis through interviews (Smith and Sparkes, 2016). For this purpose, ontological relativism and constructivist epistemology were deemed most appropriate. Ontological relativism is a philosophical framework that recognizes the inherently subjective nature of reality and assumes that reality is not an objective entity, but rather a subjective construct shaped by individual or cultural perspectives, contexts, beliefs and interactions (Schwandt, 1994). Understanding and interpreting these constructions requires interaction between the researcher and the participants. Therefore, a constructivist epistemology is naturally best suited. This epistemological stance posits that knowledge is not simply discovered or passively acquired, but is actively constructed by researchers through their interpretive engagement with participants’ perspectives, thus dynamically shaping knowledge (Guba and Lincoln, 1994).

The constructivist framework was well-suited to our research team, which included four practitioners from soccer and two researchers specializing in sport psychology. This diverse composition enabled us to approach the research from both practical and theoretical perspectives, thereby enhancing our understanding and analysis. Together, our experiences and expertise influenced the interpretation and translation of our findings.

### 2.2 Interview development

This study is part of a larger project that addresses the topic of performance crises in professional soccer (see Jekauc et al., 2024). Initially, twelve interviews were conducted with the aim of explaining underlying mechanisms of how crises emerge in professional soccer, the dynamics of a crisis, and sustaining factors that maintain a crisis. These interviews strongly highlighted the crucial role of coaches’ behavior in times of a performance crisis. Consequently, these twelve interviews were re-analyzed with a focus on the various roles coaches can adopt to manage a performance crisis. In addition, because the analysis of the initial twelve interviews emphasized the relevance of the roles coaches adapt during performance crises, four additional interviews with new coaches were conducted in a later phase to delve deeper into this specific aspect, addressing the roles of coaches during a performance crisis. Given the targeted nature of these follow-up interviews, it was determined that four additional interviews would be sufficient to gather detailed insights without redundancy, ensuring a focused and efficient use of resources.

The semi-structured interview guides for both waves of interviews were developed based on theoretical considerations [e.g., the role of the coach and the dynamics in crisis situations according to Jekauc et al. (2024)] and revised within the research team to ensure comprehensiveness and feasibility (Brinkmann and Kvale, 2018). Semi-structured interviews were chosen for their methodological coherence, which aligns with our philosophical assumptions and data analysis approach, due to their inherent flexibility.

The first draft of the interview guide for the initial twelve semi-structured interviews was pilot tested with one active coach and one active player, who were sourced from the acquaintances of the research team, in order to test the comprehensibility of the questions as well as to gain practice in interviewing (Mikuska, 2017). The coach involved in the pilot testing was not among the final sixteen coaches interviewed. Including the player in the pilot testing provided practical insights and experiences, enhancing the practice-orientation of the questions. Both the coach and the player suggested several modifications. Based on their feedback, new questions were added to further explore specific topics and address gaps in the initial draft of the interview guide.

The revised guide served the researcher as a problem-centered roadmap, allowing flexibility for exploration and a natural flow of the conversation (Witzel and Reiter, 2012). This was achieved by organizing the guide into open-ended questions, designed to explore the subjective perspective on the topic, and follow-up questions, designed to enrich the narrative. The interview guide for the initial twelve interviews focused on the emergence, dynamics, and sustaining factors of crises in professional soccer from the coaches’ perspective. The guide also included questions regarding the roles of a coach during a performance crisis and the intervention strategies they try to use. The guide can be accessed in the Supplementary materials (SM 1).

The semi-structured interview guide for the subsequent four interviews, which specifically focused on the roles coaches can adopt during performance crises, was divided into four parts. The first part was designed to get to know the participant and to build rapport by describing his own background as a soccer coach. The second part focused on the roles of a coach in general (e.g., could you tell me about the roles you fulfill as a coach and describe them in more detail?) and the third part focused on the roles of a coach during a performance crisis (e.g., could you describe a specific situation in which you have experienced a crisis and whether and to what extent your role as a coach has changed as a result?). The fourth part included a closing question in which the participants were asked if there was anything else they would like to add. The guide can be found in the Supplementary materials (SM 2).

### 2.3 Participants and interview process

Following ethical approval from the Ethics Committee of the Karlsruhe Institute of Technology, professional coaches were contacted via email or telephone and invited to volunteer for this study. A combination of purposive and convenience sampling was employed, given that the sample comprised professional coaches with limited availability. Six coaches were recruited via personal connections within the research team, while the remaining ten coaches were recruited without any prior personal contact. Importantly, the lead author did not have any personal connections with these coaches. The initial inclusion criteria were: (a) coaches must have experienced a performance crisis during their career, and (b) they must be considered professional coaches. Coaches were classified as professional if they coached a team within one of Germany’s top three divisions. However, due to accessibility, two coaches who coached the second team of a professional club were also included in the sample. The inclusion of these coaches also helped to investigate a broader range of perspectives on the topic. This procedure resulted in 16 male coaches. Their ages ranged from 32 to 54 years (*M* = 43.81, SD = 6.46), and their coaching experience ranged from 7 to 23 years (*M* = 15.44, SD = 5.0). The sample included a variety of coaching positions: ten head coaches, four assistant coaches, and two goalkeeper coaches. Additionally, the sample included coaches with various qualifications: eleven UEFA Pro Licenses, two UEFA A Licenses, one UEFA Goalkeeping A Licenses, and two coaches without an UEFA License. Furthermore, eight of these coaches were former professional players and eight played soccer on an amateur level. At the beginning of the interviews, the coaches were briefed about the study’s general purpose. Coaches were assured of confidentiality and anonymity for their answers. They were further informed that participation was voluntary and they could withdraw at any point from the study without justification. All coaches had provided written consent and agreed to the interview being audio-recorded. Two interviews were conducted in person and 14 via a digital meeting platform due to geographical constraints. The interviews were conducted in German.

The initial twelve semi-structured interviews were carried out from November 2022 to March 2023 by the lead author of the article. The duration of the interviews ranged from 39 to 97 min (*M* = 68.92; SD = 18.49). Due to the strict focus on the roles, thus a more precise and targeted inquiry, the subsequent four semi-structured interviews ranged from 19 to 21 min (*M* = 19.29; SD = 1.40) and were conducted by a research assistant in February 2024.

### 2.4 Reliability and trustworthiness

We attempted to ensure trustworthiness by prioritizing transparency, worthy topic, and resonance (Smith et al., 2014). Regarding *transparency*, we aimed to align with the twenty questions proposed by Braun and Clarke (2021) for assessing the quality of thematic analysis research (see Supplementary material SM 3). These questions address various aspects such as the rationale for employing reflexive thematic analysis, the specification of the type of thematic analysis, conceptual coherence, and the potential of the reported themes to generate actionable outcomes. The coding process was conducted in multiple ‘stages’. Initially, the lead author performed the initial coding (micro stage). Regular discussions were held with JF and DJ to review and refine the initial themes (meso stage). Subsequent fundamental discussions on the roles and themes took place with SA, JS, and LS (macro stage). This ensured that the lead author reflected on alternative interpretations and remained open-minded to alternative explanations throughout the interview and analysis process, with the research team acting as ‘critical friends’ (Smith and McGannon, 2018).

A *worthy topic* is defined as one that is “relevant, timely, significant, interesting, or evocative” (Smith et al., 2014, p. 196). We believe that our study meets these criteria, as performance crises can have wide ranging and extensive effects on all stakeholders involved (Buenemann et al., 2023; Jekauc et al., 2024). According to Braun and Clarke (2021), themes reported in applied research should lead to actionable outcomes. Consequently, the descriptions of the fundamental and soccer-specific roles provided in this study can be translated into practice, aiding coaches in managing performance crises.

Lastly, we aimed to enhance *resonance* through transferability (Tracy, 2010) and naturalistic generalizability (Smith, 2018). While transferability is achieved when the results are to some extent transferable to other contexts, naturalistic generalizability is achieved when the research connects with the reader’s personal life or vicarious experiences (Smith, 2018). In order to achieve resonance and naturalistic generalizability, Tracy (2010) suggests that researchers should “create reports that invite transferability by gathering direct testimony, providing rich description and writing accessibly and invitationally” (p. 845). We aim to offer readers vicarious experiences by presenting rich details of the participants’ behaviors during times of performance crises. In this sense, readers are encouraged to judge whether the results are transferable to other contexts and resonate with them.

### 2.5 Data analysis

All interviews were audio recorded and manually transcribed verbatim using the software f4 (dr.dresing and pehl GmbH, 3.4.5), resulting in 207 pages of single-spaced text. Reflexive thematic analysis was chosen as the methodological approach due to our belief that knowledge is inherently co-constructed. In this framework, the researcher acts as an analytic resource, actively and reflexively engaging with the data to generate themes (Braun and Clarke, 2020). Furthermore, reflexive thematic analysis is a suitable tool for identifying patterns in individuals’ behaviors concerning a specific issue. Aligning with the objective of this study, it also aids in producing research for public consumption, such as practice-oriented studies (Braun et al., 2016). Specifically, an inductive reflexive thematic analysis (Braun et al., 2016) within a broader deductive framework was employed, using the research question (i.e., What are the roles of a soccer coach during a performance crisis to manage such situations?) to guide the themes. As researchers “cannot enter a theoretical vacuum when doing TA [thematic analysis]” (Braun and Clarke, 2020, p. 4), it must be acknowledged that pre-existing theoretical assumptions inevitably biased the results. These assumptions included the definition of coaching effectiveness from Côté and Gilbert (2009) as well as literature on excellent coaching (e.g., Bloom et al., 2014; Nash et al., 2011), and performance crisis (Buenemann et al., 2023; Jekauc et al., 2024).

The analysis technique followed the recursive six-phase model proposed by Braun and Clarke (2021). Phase 1 involved data familiarization and writing familiarization notes, which was accomplished by repeatedly reading the transcribed interviews. Since the initial twelve interviews had already been coded for another study, the lead author was highly familiar with the transcripts, which facilitated the note-writing process. Phase 2, characterized by systematic data coding, was rather on a semantic level, meaning that explicitly expressed meanings and concepts were included (Braun et al., 2016). The initial themes derived from the coded data (phase 3) were continuously reviewed by examining the similarities and differences between the themes (phase 4). Phase 5 involved refining, defining, and naming themes, by including several discussions within the research group. This process resulted in hierarchically organized themes that provided an answer to the research question (Braun et al., 2016). An example of a coding process that includes phases 1 to 5 can be found in the Supplementary material (SM 4). In phase 6, during the report writing, inconsistencies, such as vague or overlapping themes, misclassifications, and redundancies became apparent. Consequently, some higher-order themes were merged and redefined, while lower-order themes were reassigned to other higher-order themes to achieve more coherent results (an example of this can be found in SM 3 – answer to question 11).

## 3 Results

### 3.1 General overview of the findings

Based on the analysis of the 16 interviews, five higher-order themes (*the Self-Manager*, *the People-Manager, the Soccer Expert, the Psychologist*, *the Administrator*) and their associated lower-order themes were generated. The findings should be interpreted as hierarchically structured. The role of the Self-Manager forms the foundation, upon which the role of the People-Manager is built, with the soccer-specific roles (the Soccer Expert, the Psychologist, the Administrator) positioned at the top. The higher-order themes of Self-Manager and People-Manager reflect fundamental roles, as they are considered essential prerequisites for soccer coaches to effectively fulfill their behaviors described in the soccer-specific roles. Specifically, the roles of the Self-Manager and People-Manager encompass fundamental competencies that help coaches understand, regulate, and influence both themselves and others. In contrast, the soccer-specific roles of the Soccer Expert, the Psychologist, and the Administrator derive from these fundamental roles and are characterized by behaviors pertinent to the soccer-specific context.

**Figure 1:**
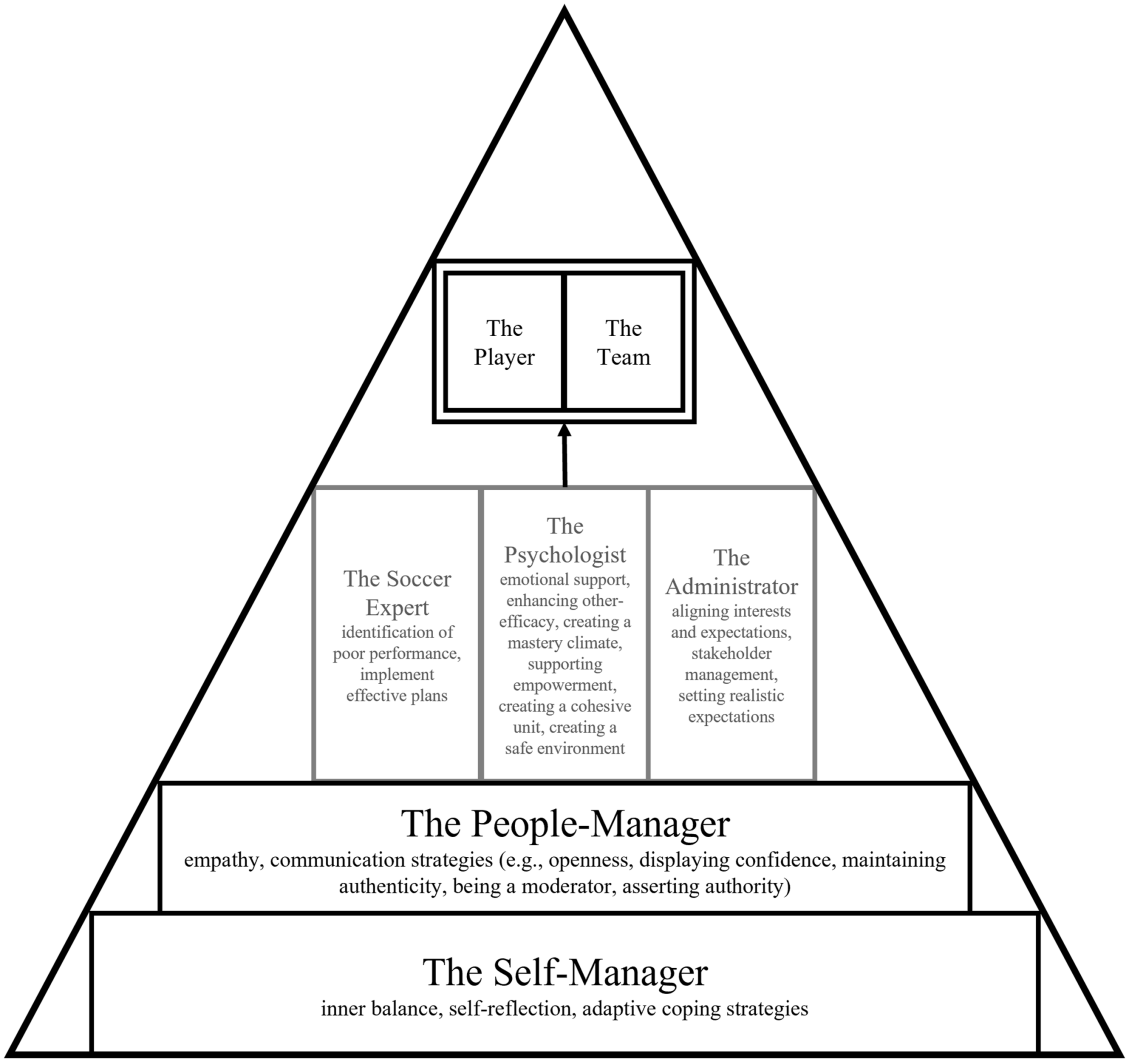
Hierarchical structure of the coaching roles. The fundamental roles are illustrated in black, while the soccer-specific roles are illustrated in grey.

Figure 1 offers a visual summary of these findings, though it simplifies the real-world complexities. The following sections explore the five high-order themes and their corresponding lower-order themes. Given that Self-Manager and People-Manager serve as fundamental roles for the soccer-specific roles, they are described first.

### 3.2 The fundamental roles

#### 3.2.1 The self-manager

The role of the *Self-Manager* involves the ability of coaches to appraise, express, regulate, and use their own emotions, behaviors and thoughts in a way that maximizes their effectiveness. This competency is fundamental, as it enables coaches to effectively fulfill the soccer-specific roles, particularly during periods of performance crises. Crucially, the role of the Self-Manager ensures coaches avoid being trapped in the downward spirals often apparent during a performance crisis, thereby retaining full access to their mental resources. Inadequately managing the processes of appraising, expressing, regulating, and utilizing their own emotions, behaviors, and thoughts may result in distorted perceptions and could ultimately lead to professional consequences, as exemplified by the experience of Coach 11:

*Yeah, I could not handle that, and it ultimately led to us parting ways. At some point, I even agreed with [name of the sports director] and I said to him ‘This does not make sense. The boys have a burden with me’, which was complete nonsense, but that’s how I saw it because I had lost my composure.*

In order to stay composed under pressure, coaches mentioned a range of techniques, which are summarized in the lower-order themes *inner balance*, *self-reflection*, and *adaptive coping strategies*. Inner balance can be regarded as the ultimate objective of the Self-Manager, with self-reflection serves as a critical prerequisite, and adaptive coping strategies represent the means to achieve, regulate, and maintain this balance.

Coaches emphasized that maintaining *inner balance* across somatic, emotional, and cognitive domains is essential for effective coaching. This balance helps avoid extreme emotional reactions, such as euphoric highs during successful periods and severe lows during challenging times. Coach 8 illustrated this issue, stating, “I always try not to be swayed by either one or the other […] We always have a detailed review for each game, and it is always the same, whether we have won or lost.” Supporting the importance of an inner balance, Coach 9 noted that he recognized a crisis “as soon as you, as a coach, feel that you have to be overly positive towards the team.” Furthermore, coaches highlighted the importance of acceptance and serenity for demonstrating an inner balance. Acceptance involves embracing challenging periods instead of resisting or avoiding them, and perceiving crises as opportunities for growth and learning as Coach 1 emphasized: “I fundamentally believe that all circumstances serve us for the best. Therefore, I actually accept the conditions that are unfavorable for a long time.” Serenity, another expression of inner balance, was stressed by Coach 11:

*Not only must you not lose your cool in a crisis situation, but you also can’t start seeing conspiracies or bad intentions in every comment or action. Sure, that kind of stuff will happen a lot, but if you start focusing only on those things, you’ll just be driven by them. Do your job 100 percent, stay calm, and focus on what you can control. The rest, you can’t change anyway.*

A prerequisite for achieving this inner balance is *self-reflection*. Coaches highlighted the importance of continuous self-reflection throughout the coaching process to ensure that the efficacy of their regulation strategies are effective. This iterative process helps in identifying not only tactical adjustments but also in understanding the broader impact of their leadership style on the players, ultimately ensuring they remain effective under pressure. As Coach 11 noted:

*Of course, as coaches, we always have to reflect on things: Are we working optimally? Are we analyzing it correctly? Are we providing the right plan? Do the expectations or tasks or what the coach wants align with what the team shows?*

*Adaptive coping strategies* (Lazarus and Folkman, 1984) are crucial in achieving, regulating, and maintaining inner balance. Coaches highlighted the use of various techniques to this end, broadly categorized into problem-focused and emotion-focused coping strategies. Regarding the latter, Coach 4 suggested that emotions are often a “burden” and that becoming aware of and regulating one’s emotions could lead to better decisions and improved cooperation in stressful situations: “I think emotion regulation, being aware of your own emotions, can be really helpful. I believe it’s extremely important when taking action together with others in a crisis situation.” Coaches in this study achieved this emotion regulation by various strategies. While Coach 10 resorted to reading literature on cognitive processes, psychology, and Buddhism as a coping strategy, Coach 12 sought support from peers and family in addition to engaging in personal activities to avoid being overwhelmed by the crisis. With regard to problem-focused coping strategies, the coaches emphasized the importance of adopting a mindset that is characterized by striving for solutions rather than ruminating on uncontrollable external events. For instance, Coach 12, who took over the club mid-season following the dismissal of the former head coach, described conducting a SWOT analysis of the club, administering a personality test for the team, and developing a communication plan.

*First, we conducted a team analysis: What types of players do we really have in this team to overcome the crisis, what are the strengths and weaknesses of each individual? We reassessed the game philosophy, ‘How do we actually want to play with this team? Can they even implement the possession style they played before?’ Then, of course, we started the communication, ensuring that we didn’t leave the team alone. (…) We had to communicate these issues and get everyone back on board, saying (…) ‘We need every employee, we need everyone from the board, we really need everyone to look positively forward.’ And we also met with the fans, because they were, of course, a bit skeptical about everything (…) and we said ‘We need you,’.*

#### 3.2.2 The people-manager

The role of the *People-Manager* refers to the process of leading, guiding, and supporting individuals or teams to achieve collective goals while fostering a positive environment. Although coaches play a central role, they prefer to maintain an equal standing with the players. As Coach 8 articulated, “I rarely want to put myself in the spotlight. Or somehow place myself at the center, because the center, the core, is the team and the club.” Based on this collaborative approach, coaches are responsible for aligning the bigger picture with collective responsibility and thus maintaining a high level of effort: “So, we subordinate everything again; it’s about the bigger picture. Everyone has to contribute to the bigger picture. No one is exempt” (Coach 9). During periods of performance crises, which are often marked by dynamic and challenging conditions, people-management competencies are necessary in order to understand human behavior, motivate individuals, and create conditions where everyone -including players and board member- feel engaged and valued. The associated lower-order themes are *empathy* and *communication strategies*, which are essential for recognizing the needs of individuals and influencing them in a meaningful way.

First, coaches emphasized the importance of *empathy* in comprehending and discerning the needs of others as a foundation for establishing meaningful interpersonal relationships. By focusing on the needs of others and demonstrating full, undivided presence, coaches reinforce their value and cultivate deeper, more authentic connections. Coach 16 elaborated on this point by stating:

*Empathy or, yes, I would probably call it empathetic listening, really listening. And there’s no phone on the table, no Apple Watch involved, but it’s really about, ‘I’m listening to you now because it’s important to me.’ (…) Truly listening. Yes, I need that because through this listening, a connection with the player naturally develops.*

Besides understanding and perceiving the needs of others, coaches highlighted the importance of profound *communication strategies* in order to guide them and fulfill the soccer-specific roles. Here, the coach acts as the central figure who has to balance various demands, including individual needs, team dynamics, staff input, and organizational goals. As Coach 13 emphasized, “When you are in professional sports, you are actually the one where all the pieces fit together, and you have to be able to communicate.” In this context, the effectiveness of communication strategies varies depending on the circumstances and the individuals involved. For instance, when dealing with distressed players, it is more beneficial to be an empathic listener rather than relying on authoritative measures to influence them. Key strategies include open communication, displaying confidence, maintaining authenticity, being a moderator, and asserting authority.

Coaches emphasized the significance of open communication during performance crises for various reasons. On one hand, it serves as a means to create an environment where players can freely express their emotions. As Coach 6 noted, “Engaging in frequent conversations with them [the players], providing them with a sense of security, and allowing them to express their fears or frustrations. It’s about sharing these moments together and simply exchanging thoughts.” On the other hand, open communication can offer clarity and stability by providing feedback and clarifying expectations as expressed by Coach 6: “We quickly found ourselves in a high-pressure situation. In that context, I tried to communicate clearly what I expected from them. This was meant to provide clarity, stability, and also a bit of strength.”

Coaches emphasized the importance of displaying confidence. Considering that “the players look to you for guidance “(Coach 6), Coach 2 highlighted that his head coach, despite occasionally facing uncertainty, consistently demonstrated a winning mentality and confidence in front of the team, which might reassure the players and instils a sense of belief: “There are moments when he has doubts, but that’s more in the coach’s office. But as soon as he approaches the team, you can see how he suddenly begins to express confidence and be like: ‘I have no doubts’.”

Maintaining authenticity was mentioned by the coaches as a tool of building trust and commitment. “Authenticity is definitely important. I think it’s a killer if you are not authentic anymore because then you lose the whole team.” (Coach 10). Alibi actions, such as benching a player just to make a statement rather than for performance-related reasons, could quickly backfire as they would undermine trust and commitment. These symbolic actions can be perceived as unfair or arbitrary, leading to a lack of confidence in the coach’s leadership.

Being a moderator can be beneficial when engaging with stakeholders, such as the board. In this role, coaches should aim to facilitate a shift in perspective among stakeholders. This is illustrated by Coach 4, who emphasized the importance of keeping the executive board informed to enable them to see the bigger picture and make more informed decisions. In this context, being a moderator ensures that decisions are based on comprehensive and accurate information, thereby fostering a more stable environment: “I also moderate, for example with the sports director or the president, to give insights from the team and put things in perspective. So, enabling a change of perspective through me (…). I want (…) to keep everyone up to date. (Coach 4).”

Lastly, coaches emphasized that asserting authority by making harsh and difficult decisions is sometimes necessary if it benefits the collective well-being. As Coach 1 stated, “I believe that leadership is paradoxical. You have to build relationships, but at the same time, if it’s for the greater good, you have to end them.” This paradox highlights the fine balance coaches must maintain between fostering strong interpersonal connections and making tough decisions that serve the team’s best interests.

### 3.3 The soccer-specific roles

As mentioned earlier, the fundamental roles enable coaches to appraise, express, regulate and use their own emotions, behaviors and thoughts and to lead, guide, and support others, which allows to perform the following roles in an effective manner. These roles are characterized by behaviors that support coaches in leading the team through a performance crisis in the context of professional soccer. Importantly, these roles should not be viewed as discrete or independent. Instead, they function as interdependent components within an integrated framework.

#### 3.3.1 The soccer expert

The role of *the Soccer Expert* is characterized by the possession of soccer-specific knowledge and expertise, encapsulated by the principle of being ‘all about the ball’. The lower-order themes are *identification of poor performance* as well as *development and implementation of effective plans*.

The *identification of poor performance* is a critical function of the Soccer Expert as highlighted by Coach 10: “Well, of course, from a sporting perspective, you have to look at: Why aren’t you achieving the results? What is the reason? Analyzing the whole situation. Are there tactical reasons or is it a matter of quality?.” This diagnostic capability allows coaches to disentangle the multifaceted causes of suboptimal performance, whether they stem from tactical errors, individual skill deficiencies, or broader systemic issues.

Moreover, the *development and implementation of effective plans* is a hallmark of the Soccer Expert. This involves making tactical adjustments during matches and designing tailored training programs. These behaviors provide players with a structured framework to focus on the task rather than outcomes:

*The first game was in [name of the club] and then he prepared the team specifically for the opponent, analyzed the opponent, tailored the training to the opponent because he knew: ‘Okay, we want to incorporate these specific elements against [name of the club], so we have to train them,’ and then we trained them. (Coach 2)*

At the same time, coaches highlighted the importance of simplicity, particularly during periods of performance crisis. Coach 6 underscored the notion that sometimes, emphasizing fundamental skills and principles can be more effective than focusing on complex techniques. This ‘less is more’ approach can be instrumental in rebuilding players’ self-confidence during a performance crisis and demonstrates how the roles complement each other. “But primarily, it’s about working on the basics again and meeting the players where they are, to give them back a bit of trust. Providing confidence, because that is completely lost in a crisis.” (Coach 6).

#### 3.3.2 The psychologist

The role of *the Psychologist* emphasizes the importance of understanding and addressing the psychological needs of both individual players as well as the team as a collective. This role is especially important in times of a performance crisis, where both interpersonal and intrapersonal challenges arise. By adopting the role of a psychologist, coaches can assist their players in managing pressure, building self-confidence, and fostering a constructive atmosphere. While specific actions might appear to predominantly benefit either an individual player or the broader team, it is crucial to recognize that the interplay between individual behaviors and team dynamics is highly interconnected. During performance crises, these reciprocal influences mean that psychological support for the individual player invariably contributes to overall collective well-being, and vice versa. Therefore, it is neither feasible nor practical to disentangle the psychological interventions aimed at the individual from those directed at the team. The associated lower-order themes for the psychologist are *emotional support*, *enhancing other-efficacy*, *creating a mastery climate*, *supporting empowerment*, *creating a cohesive unit*, and *creating a safe environment*.

Coaches have reported that offering *emotional support* involves behaviors that cultivate a sense of affiliation, thereby fostering strong interpersonal relationships. This is perceived as fundamental by the coaches, as expressed by Coach 7: “That you build an emotional connection with them [the players], that you get along well with them on a personal level, that’s the most important thing.” Behaviors such as demonstrating understanding and concern or offering a shoulder to cry on are intended to help players in overcoming the adverse psychological processes that emerge in times of a performance crisis. Coach 8 illustrated this by stating:

*When you’re not doing so well, what do you want more than ever? Security, strength, someone who still believes in me, someone I can lean on, maybe on a more relationship level, who gives me a shoulder to lean on or something. But if you transfer all that to the sport, that’s all we’ve done.*

*Enhancing other-efficacy* (Bandura et al., 1980) emphasizes that coaches should try to enhance the players’ self-confidence by demonstrating their belief in the players and the team. Coach 12 illustrated this by describing how he and his head coach discussed how to approach the team, which was on the verge of relegation, before this all-important final:

*He [the head coach] said, ‘Yeah, I wouldn’t say much tactically, just some motivating words,’ and I was like, ‘Hey, wait a minute, let’s give this moment to the players. Let’s just step back and let them have this moment.’ So, I told [name of the head coach], ‘I’ll make a video and show them what they’ve achieved.’ This way, they can really see what they’ve accomplished up to this point, allowing us to play in this final.*

*Creating a mastery climate* (Duda and Balaguer, 2007) describes that instead of being driven by the urgency or pressure of an upcoming match and focusing solely on results, coaches should strive for development. For example, as Coach 2 explained, his head coach focused on defensive compactness, thus avoiding putting additional pressure on the players by emphasizing the crisis situation: “He focused on specific training content: ‘Today, we are training defensive compactness, today we are working on offensive principles, today we are improving our counter-pressing. […] So, he simply focused on content-related issues and less on this crisis situation” (Coach 2). Even though performance crises are closely related to negative results, the coaches stressed the importance of focusing on the development of the players rather than focusing on outcomes.

*The results were always just a consequence of my efforts to make everyone better. I didn’t put too much emphasis on the results because I believe that if we work well, if we try to make everyone better, the results will come automatically. (Coach 9)*

This mastery climate could further be reinforced by *supporting empowerment* (Deci and Ryan, 1987) involving the players in decision-making processes. Coach 12 stated, “that every employee should feel appreciated, competent, and acknowledged.” In this context, Coach 8 stated that coaches are dependent on the players and therefore should strive to involve the players in the decision-making processes: “But it’s a constant exchange. I can set up the framework in a way that works great for me. But I need the players to be able to identify with this framework as well.”

*Creating a cohesive unit* (Carron et al., 2002) even in times of a performance crisis is essential. This is also exemplified by Coach 3, who asserted that a stronger sense of team cohesion enhances the ability to manage and overcome external adversities. He illustrated this by drawing a circle to represent the team and its support system, including the coaching staff, players, and manager. Subsequently, he drew arrows to symbolize external pressures, such as criticisms from fans, media scrutiny, and poor results, which attempt to weaken the bond within this inner circle. His explanation for this drawing was:

*To make it clear to them how important this inner circle is. How important it is to stick together as a team, not just players, but also the coaching staff, the whole team, to deal with everything that comes from outside, because adversities come from outside, through influences, personal influences, or fans, or press, or media. And that these adversities can only be overcome if the team, the inner circle, comes even closer together than they already are.*

To establish a strong sense of cohesion, coaches emphasized the importance of *creating a safe environment* (Edmondson, 1999). They used various strategies to achieve this. For instance, Coach 8 highlighted the significance of ensuring that “no one was put on the spot,” stressing the importance of not pointing out individuals. Furthermore, Coach 4 emphasized the significance of mutual respect by stating that “even though we have lost a game, there’s no one acting like a jerk in the locker room. We’re all still sportsmen.”

Additionally, coaches frequently mentioned the need to protect the team from external pressure to foster trust and security among players. For example, Coach 1 stated:

*There will be two or three defeats, and this pressure is then simply passed on to the team, unfiltered. And filtering that would be the task of a good leader. So that players and staff feel secure, even though it may be unfavorable for achieving goals for a long time.*

#### 3.3.3 The administrator

The role of *the Administrator* is characterized by managing and coordinating the administrative and organizational elements, which are crucial for maintaining team functionality, particularly during performance crises. It requires coaches to adopt a multidirectional perspective, attending to the needs of various stakeholders, including players, staff, and board members, while navigating the complexities that arise in high-pressure environments. This theme includes the lower-order themes *aligning interests and expectations*, *stakeholder management*, and *setting realistic expectations*.

During a performance crisis, *aligning interests and expectations* becomes critical, especially since the executive board, which ultimately decides the coach’s employment, may act impulsively under pressure. As Coach 11 noted, “If the executive board, the sports manager, and the overall management are in agreement and remain calm, then such situations can be well managed and these crises can be overcome. If the situations are assessed differently, then it becomes tricky.”

*Stakeholder management* highlights the necessity to keep all stakeholders involved and to bridge the gap between various stakeholders within the club. According to Coach 1, coaches “must try to understand all the viewpoints, perceptions, and interests of those involved.” This is particularly important given that stakeholders often have diverse professional backgrounds, which can lead to differing perspectives as explained Coach 8:

*This [intensive communication] is important to me because often, these are not people who have actively played soccer themselves, but they come from the business world and are often executives. So, we need to collaborate, even on questions like, what do you do when things aren’t going well in your company, and how can I apply that to sports? Tell me about the differences.*

Finally, *setting realistic expectations* and adjusting these expectations is essential to navigate difficult phases as highlighted by Coach 11:

*Yes, healthy structures essentially mean a realistic and objective approach. When we talk about crises and how to view things during such times. By healthy structures, I mean a factual and realistic examination of the current situation: Why are we currently unable to meet expectations? Why is the team not achieving the points as hoped? If you assess this realistically, identify and name the reasons, and the club says, ‘Yes, that makes sense, we can understand that,’ then nothing happens, and you get through these phases as well.*

## 4 Discussion

The purpose of the present study was to explore the different roles that coaches adopt to manage performance crisis in professional soccer. Through qualitative interviews with experienced coaches, the study identified both fundamental and soccer-specific roles. These roles are organized hierarchically, with fundamental roles serving as a foundation for soccer-specific ones. Importantly, the hierarchical structure of these roles is not just relevant from a theoretical perspective, but it also has practical implications. Soccer coaches must first master the fundamental roles of the Self-Manager and the People-Manager before they can effectively fulfill the soccer-specific roles. For instance, without mastering the Self-Manager role, even the most skilled Soccer Expert may struggle to implement effective training sessions if the coach loses their inner balance due to various stressors. This can result in the coach passing the pressure they experience directly onto the team, thereby undermining performance. Specifically, composure is required in the role of the Soccer-Expert to maintain focus, make strategic decisions, and provide clear instructions during high-pressure situations. A lack of composure can lead to poor decisions and a loss of confidence among players, which can have a negative impact on team performance. Similarly, the role of the Psychologist can only be effectively fulfilled if the coach has understood the principles of the People-Manager role, as empathy is a prerequisite for addressing the needs of their players. Therefore, adapting communication methods to the specific needs of stakeholders ensures effective interaction and collaboration. Furthermore, these roles represent interdependent components through which coaches provide holistic support to their players. For example, boosting player’ self-confidence involves the Soccer Expert’s tailored matchday preparation and the Psychologist’s role in fostering psychological safety. This integrated approach highlights the importance of combining various coaching behaviors to address individual players’ needs and build team resilience during challenging situations.

### 4.1 The self-manager

The higher-order theme of the Self-Manager was generated to represent a fundamental role in times of a performance crisis. The Self-Manager corresponds with the intrapersonal knowledge component in Côté and Gilbert’s (2009) conceptualization of coaching effectiveness, as it embodies the ability to review, revisit, and reflect on one’s actions. As Côté and Gilbert (2009) postulated, the composition of effective coaches’ professional, interpersonal, and intrapersonal knowledge should vary according to the coaching context. The present study supports this postulate, emphasizing that intrapersonal knowledge is fundamental in the applied coaching context of a performance crisis, as it enables subsequent coaching roles. Effective self-management competencies are crucial for coaches navigating performance crises. This is further supported by a survey conducted among U.S. Olympic coaches, which revealed that those who struggled to manage crisis situations perceived themselves as ineffective (Gould et al., 2002). Conversely, coaches who remained composed under pressure perceived themselves to be able to effectively coach their athletes and teams.

Given that elite coaches consistently operate in highly stressful environments (Chroni et al., 2016; Olusoga et al., 2009; Thelwell et al., 2008), the present study’s participants emphasized the importance of achieving inner balance during performance crises, aligning with the findings of Lara-Bercial and Mallett (2016). Various external stressors can disrupt this inner balance, making self-reflection and profound coping mechanisms, both emotion-focused and problem-focused, essential for coaches to maintain their inner balance. A construct that could support coaches in achieving their inner balance is emotional intelligence, defined as the ability to appraise, express, regulate and use emotions (Salovey and Mayer, 1990). Emotional intelligence has been shown to enhance effective coping in the face of challenges, support decision-making, and foster positive interpersonal relationships (Chan and Mallett, 2011; Jowett et al., 2024).

### 4.2 The people-manager

The present study identified the role of the People-Manager as another fundamental role, reflecting the interpersonal knowledge component in Côté and Gilbert’s (2009) conceptualization of coaching effectiveness. Profound empathy and communication competencies can facilitate effective interactions, thereby enhancing communication with various stakeholders. The existing coaching literature underscores a set of competencies and skills for effective coaching. Notably, Lara-Bercial et al. (2017) describe in their European Sport Coaching Framework coaching competencies with regard to six primary coaching functions (e.g., build relationships, shape the environment). However, unlike Lara-Bercial et al. (2017), the present study focuses on fundamental competencies that are crucial across all soccer-specific behaviors described in the roles, rather than assigning specific competencies to each behavior. Considering that coaches need to adapt their behaviors to different circumstances (Lara-Bercial et al., 2017), the present study suggest that during performance crises, it is particularly essential to possess profound communication strategies and empathy.

The communication strategies described in the present study are further consistent with the COMPASS model developed by Rhind and Jowett (2010) for relationship maintenance strategies in the coach-athlete relationship. This model includes key elements such as conflict management, openness, motivation, positivity, advice, support, and social networks that are instrumental in maintaining high-quality relationships and athlete’s experiences of sport satisfaction (Davis et al., 2019). Furthermore, similar to the findings of Lara-Bercial and Mallett (2016), coaches reported employing a combination of cooperative and directive approaches, adjusting their methods based on situational demands and individual players’ needs. For instance, exerting pressure on players to achieve better results may be counterproductive if the players are already struggling with the demanding nature of a performance crisis. Nonetheless, the findings of the present study indicate that asserting authority is sometimes necessary, but it must be applied with authenticity and always with the team’s overall well-being in mind, aligning with the qualitative investigation of highly successful Olympic coaches by Lara-Bercial and Mallett (2016).

### 4.3 The soccer expert

During the periods of performance crises, the coaches highlighted the importance of soccer-specific expertise, which defines the role of the Soccer Expert. In relation to Côté and Gilbert’s (2009) framework of coaching effectiveness, the role of the Soccer Expert exemplifies the component of professional knowledge. However, as coaches in this study reported, in times of a performance crisis, it is more important to cover the “basics” rather than to implement extraordinary training sessions. For this reason, the results of the present study suggest that while professional knowledge is crucial for identifying the reasons for poor performance and establishing effective plans (Cook et al., 2021), its importance during a performance crisis extends beyond technical aspects to include psychological support. As players are already struggling with the demanding nature of the crisis, this expertise is leveraged not only to improve players’ skills but also to rebuild their self-confidence and establish a framework that emphasizes a task-oriented focus over an outcome-oriented focus (Duda and Balaguer, 2007).

As Jowett and Shanmugam (2016) noted, technical proficiency is essential, but what truly gives coaches the “edge” is the connection developed between the coach and the athlete. While recognizing the needs of their players is valuable, this alone may not suffice without professional expertise at a professional level. Conversely, pure technical knowledge without the ability to recognize the needs of their players is also inadequate. To successfully navigate performance crises, coaches must possess soccer-specific expertise as well as the ability to build strong interpersonal relationships with their players, aligning with previous research on managing challenging situations (Olusoga et al., 2012; Slade et al., 2024; Zhao and Jowett, 2022).

### 4.4 The psychologist

The behaviors identified in the role of the Psychologist contribute to building strong relationships between the coach and the players as well as fostering a mastery climate. Jowett and Shanmugam (2016) describe the relationship between coaches and players as the “heart of effective and successful coaching” (p. 472). In fact, Jowett argues that the quality of the coach-athlete relationship encapsulates the essence of coaching effectiveness described by Côté and Gilbert (2009). Effective coaching depends on the quality of the relationship and encompasses essential elements such as respect, trust, commitment, and collaboration (Jowett, 2017). These elements are vital for fostering positive and mutual influence which, in turn, encourages athletes to accept coaching.

During performance crises, it is crucial to focus on the players as an individual, not just their performance. This may seem counterintuitive at first, given that performance crises are closely linked to negative results and the high emphasis on outcomes in professional sports. However, as Jekauc et al. (2024) suggest, psychological processes at both the individual (e.g., anxiety, reduced self-confidence) and team levels (e.g., reduced cohesion, conflict) often contribute to declining performance. Therefore, emphasizing the interpersonal relationship and fostering a mastery climate may be especially helpful in times of a performance crisis. In fact, research consistently shows that a strong coach-athlete relationship improves performance, satisfaction, motivation, persistence, team cohesion, and collective efficacy (Jowett and Shanmugam, 2016). Additionally, perceptions of a mastery climate are linked to adaptive motivational outcomes such as competence, self-esteem, objective performance, affective states, and intrinsic forms of motivational regulation (Harwood et al., 2015), which are crucial for navigating performance crises.

### 4.5 The administrator

During performance crises, the actions of the board and the pressure faced by coaches underscore the importance of the Administrator’s role. Two key aspects could be crucial during such times: the principle of subjectivity and organizational memory. The principle of subjectivity means that how individuals perceive a crisis depends largely on their interpretation (Kleinert, 2003). Crises are inherently part of a social system (Kleinert, 2003), affecting different individuals in various ways based on their perceptions. For example, one individual may perceive the situation as a crisis, while another may see it as merely a downhill slide. Similarly, a crisis situation for one individual can affect different actors, making crises social processes influencing a diverse range of stakeholders. To effectively resolve such situations, all involved parties should share a similar understanding of the situation, a process known as reality negotiation (Higgins and Leibowitz, 1999). From this perspective, aligning interests and expectations as well as stakeholder management are essential during crises to protect the team and guide the organization effectively.

The need for this consideration is further supported by findings demonstrating how stakeholders outside a football club challenged internal operations and how pressure and expectations from external surroundings affected the football team and its members (Bjørnstad et al., 2024; Horton, 2014). In this sense, organizational memory functions as an “almost-magnetic force,” creating a pull towards past practices, which leads to rigidity in decision-making and hinder adaptability (Bjørnstad et al., 2024). To counter this, promoting open communication, managing conflicts, and encouraging feedback are vital for maintaining flexibility and fostering a positive organizational culture (Bjørnstad et al., 2024).

### 4.6 Limitations and future research

Several limitations of the study should be taken into consideration. One notable limitation is the narrow sample, which was restricted to male coaches, focused solely on soccer, and included only individuals based in Germany. This may have biased the perceived importance of certain roles and behaviors during performance crises. Additionally, the study only included interviews with coaches, thereby overlooking the perspectives of players and other stakeholders. This is significant because the behaviors deemed effective by coaches may not align with the players’ needs during performance crises (for an overview of the players’ needs, see Sousa et al., 2024b). Therefore, future research should include a broader sample encompassing various stakeholders and genders to identify strategies tailored to the diverse needs of both coaches and players. Another limitation is that the coaches described their experiences with performance crises and behaviors retrospectively. Consequently, a long-term ethnographic study of a club in crisis could provide deeper insights into effective behaviors and stakeholder interactions. Additionally, the findings may be influenced by the specific contexts of the interviewees. The inclusion of two coaches from the second team of a professional club helped to investigate a broader range of perspectives but also influenced the analysis, as these coaches work with players who have different profiles and ambitions compared to more established players. Future studies could benefit from examining the roles and perspectives of coaches working with different player profiles to provide a more comprehensive understanding of how various coaching contexts influence the coach’s responsibilities.

Based on the findings, we propose several directions for future research. First, while defining a specific context -such as a performance crisis in professional soccer in the present study- is crucial for addressing the specific external demands (Lyle, 2002), exploring other contexts by examining various sports would broaden our perspective. Second, understanding how players from different cultural backgrounds cope with crises can enhance coach support. Third, following Wu et al. (2021), crises can be separated into pre-crisis, crisis, and post-crisis phases. Recognizing that different measures may be more effective at various phases (Zhao and Jowett, 2022), future research could categorize the generated behaviors as preventive, managing, or reflective and empirically investigate the effectiveness of these behaviors. Furthermore, future studies could investigate different challenging situations. For instance, dealing with injuries differs from handling periods of poor results or conflicts within the management, but all these scenarios can significantly influence a coach’s responsibilities. Understanding how different scenarios affect the decision-making process could provide valuable insights into coaching strategies and adaptability, specifically how coaches could adjust their approach based on specific challenges.

### 4.7 Applied implications

The findings offer several significant practical implications for coaches dealing with performance crises. Because performance crises are almost an inevitable aspect of professional sports, preparation is crucial in order to maintain an inner balance and navigate effectively through such a time. Chroni et al. (2016) suggested that preparation could mitigate the negative effects of the various stressors on coaching effectiveness, with coping skills learned through excellent planning, reflection, and a flexible mindset. Applied to performance crises, this could involve: (a) understanding the mechanisms and consequences of performance crises (Buenemann et al., 2023; Jekauc et al., 2024), (b) adopting adaptive coping strategies, (c) proactively participating in emotional intelligence training interventions (e.g., Campo et al., 2016), and (d) participating in mental skills training programs like “Coaching under Pressure” for building resilience (e.g., Olusoga et al., 2014).

Effective communication can be seen as the means for sustaining a strong coach-athlete relationship (Jowett and Shanmugam, 2016), as well as a key factor for managing and preventing performance crises (Kleinert, 2003). The COMPASS model by Rhind and Jowett (2010) could provide coaches with a framework for effective communication, enabling them to understand and manage the complex dynamics of the coach-athlete dyad. Coaches should therefore be encouraged to establish effective communication strategies. Furthermore, given that coaches operate within a broader organizational framework, they should adopt a multidirectional communication approach. Coaches are encouraged to engage effectively with those who operate alongside the coach (e.g., staff), above the coach (e.g., board members, sports manager), and in parallel to the coach (e.g., fans, media; Cruickshank and Collins, 2015). Such a comprehensive communication strategy not only promotes transparency and understanding within the team but also strengthens relationships with key stakeholders outside the coach’s immediate environment.

Many of the behaviors identified for managing performance crises could also be effectively utilized as preventive measures. Coaches in the study emphasized the importance of building a trusting coach-athlete relationship, creating a mastery climate, fostering a psychologically safe environment, employing an empowering leadership style, and maintaining an inner balance. These approaches align with the principle of driven benevolence found in highly successful coaches, which is characterized by a persistent and balanced desire to support oneself and others considerately (Lara-Bercial and Mallett, 2016). Given that these behaviors are generally effective with regard to performance (Lochbaum et al., 2022), regardless of whether a crisis is present, coaching education programs should place greater emphasis on sport psychology techniques. Additionally, these programs can support coaches in exploring their values and beliefs and in developing a personal, ethically grounded coaching philosophy based on the principle of driven benevolence.

Historically, the evaluation of coaches has relied heavily on performance outcomes (Nash and Mallett, 2019), especially in soccer, where board members are not directly involved in the coaching process. Coaches often feel judged solely by their teams’ results. However, Côté and Gilbert (2009) argue that this is an inadequate measure of coaching effectiveness. Effective coaching should include not only team and individual performance but also the coach-athlete relationship and athlete outcomes (competence, confidence, connection, and character). In line with this, Buchheit et al. (2023) redefined the performance assessment of staff in elite organizations through a survey involving 51 practitioners. They found that highly relevant indicators focus on communication, collaboration, self-reflection, and personal growth, which align with the findings of the current study. The absence of a broader perspective may account for the high turnover rate of coaches in professional soccer. Board members who focus solely on immediate results may neglect the significance of interpersonal relationships and athlete development. Even if performance crises are associated with periods of failure, dismissing the coach could be detrimental to long-term success, as it undermines the coach-athlete relationship, which is central to effective coaching (Jowett, 2017; Nash and Mallett, 2019). Therefore, it is essential for all stakeholders to understand and apply the core principles of coaching effectiveness.

## 5 Conclusion

The present study examined the roles coaches adopt to manage performance crises. By identifying fundamental roles (Self-Manager and People-Manager) that enable the soccer-specific roles (Soccer Expert, Psychologist, and Administrator), the findings highlight how coaches combine a range of interdependent behaviors to support athletes’ needs and maintain team resilience. Although this research offers new insights into effective crisis management, its retrospective nature and relatively homogenous sample call for broader, more diverse investigations using varied methodologies. Overall, this study contributes to the growing body of literature on effective crisis leadership in elite sports and provides practical implications to guide coaches in challenging times.

## Data Availability

The raw data supporting the conclusions of this article will be made available by the authors, without undue reservation.

## References

[ref1] Bandura A. Adams N. E. Hardy A. B. Howells G. N. (1980). Tests of the generality of self-efficacy theory. Cogn. Ther. Res. 4, 39–66. doi: 10.1007/BF01173354

[ref2] Benz M. Gehring S. (2009). Krisen im Profifußball: Chancen und Herausforderungen für Lizenzgeber und Insolvenzverwalter. Stuttgart: Boorberg.

[ref3] Bjørnstad M. Tam A. McDougall M. Feddersen N. B. (2024). Relationships influencing organisational culture in men’s elite football clubs in Norway. Psychol. Sport Exerc. 72:102604. doi: 10.1016/j.psychsport.2024.102604, PMID: 38316334

[ref4] Bloom G. A. Falcão W. R. Caron J. G. (2014). “Coaching high performance athletes: implications for coach training” in Positive human functioning from a multidimensional perspective. eds. Gomes A. R. Resende R. Albuquerque A., vol. 3 (Nova Science Publishers), 107–131.

[ref5] Braun V. Clarke V. (2020). One size fits all? What counts as quality practice in (reflexive) thematic analysis? Qual. Res. Psychol. 18, 328–352. doi: 10.1080/14780887.2020.1769238, PMID: 40101104

[ref6] Braun V. Clarke V. (2021). Can I use TA? Should I use TA? Should I not use TA? Comparing reflexive thematic analysis and other pattern-based qualitative analytic approaches. Couns. Psychother. Res. 21, 37–47. doi: 10.1002/capr.12360

[ref7] Braun V. Clarke V. Weate P. (2016). “Using thematic analysis in sport and exercise research” in Routledge Handbook of Qualitative Research in Sport and Exercise, eds. Smith B. Sparkes A. C. (London: Routledge), 1, 191–205.

[ref8] Brinkmann S. Kvale S. (2018). Doing interviews, vol. 2. London: SAGE Publications Ltd.

[ref9] Buchheit M. Schuster L. King R. (2023). Beyond the scoreboard: redefining performance staff assessment in elite sports organizations. Sport Perf. Sci. Rep. 1, 1–10.

[ref10] Buenemann S. Raue-Behlau C. Tamminen K. A. Tietjens M. Strauss B. (2023). A conceptual model for performance crises in team sport: a narrative review. Int. Rev. Sport Exerc. Psychol., 1–26. doi: 10.1080/1750984X.2023.2291799, PMID: 40101104

[ref11] Campo M. Laborde S. Mosley E. (2016). Emotional intelligence training in team sports. J. Individ. Differ. 37, 152–158. doi: 10.1027/1614-0001/a000201, PMID: 39599714

[ref12] Carron A. V. Colman M. M. Wheeler J. Stevens D. (2002). Cohesion and performance in sport: a meta analysis. J. Sport Exerc. Psychol. 24, 168–188. doi: 10.1123/jsep.24.2.168

[ref13] Chan J. T. Mallett C. J. (2011). The value of emotional intelligence for high performance coaching. Int. J. Sports Sci. Coach. 6, 315–328. doi: 10.1260/1747-9541.6.3.315

[ref14] Chroni S. A. Abrahamsen F. Hemmestad L. (2016). To be the eye within the storm, I am challenged not stressed. J. Appl. Sport Psychol. 28, 257–273. doi: 10.1080/10413200.2015.1113449, PMID: 40101104

[ref15] Cook G. M. Fletcher D. Carroll C. (2021). Psychosocial functioning of Olympic coaches and its perceived effect on athlete performance: a systematic review. Int. Rev. Sport Exerc. Psychol. 14, 278–311. doi: 10.1080/1750984X.2020.1802769

[ref16] Côté J. Gilbert W. (2009). An integrative definition of coaching effectiveness and expertise. Int. J. Sports Sci. Coach. 4, 307–323. doi: 10.1260/174795409789623892

[ref17] Cruickshank A. Collins D. (2015). “The sport coach” in Leadership in sport. eds. O’Boyle I. Murray D. Cummins P. (Abingdon, Oxon: Routledge), 155–172.

[ref18] Davis L. Jowett S. Tafvelin S. (2019). Communication strategies: the fuel for quality coach-athlete relationships and athlete satisfaction. Front. Psychol. 10:2156. doi: 10.3389/fpsyg.2019.02156, PMID: 31607989 PMC6770846

[ref19] Deci E. L. Ryan R. M. (1987). The support of autonomy and the control of behavior. J. Pers. Soc. Psychol. 53, 1024–1037. doi: 10.1037//0022-3514.53.6.1024 3320334

[ref20] Duda J. Balaguer I. (2007). “The coach created motivational climate” in Social psychology in sport. eds. Jowett S. Lavallee D. (Champaign, IL: Human Kinetics).

[ref21] Edmondson A. (1999). Psychological safety and learning behavior in work teams. Adm. Sci. Q. 44, 350–383. doi: 10.2307/2666999

[ref22] Gómez M. A. Lago-Peñas C. Gómez M. T. Jimenez S. Leicht A. S. (2021). Impact of elite soccer coaching change on team performance according to coach- and club-related variables. Biol. Sport 38, 603–608. doi: 10.5114/biolsport.2021.101600, PMID: 34937970 PMC8670813

[ref23] Gould D. Greenleaf C. Guinan D. Chung Y. (2002). A survey of U.S. Olympic coaches: variables perceived to have influenced athlete performances and coach effectiveness. Sport Psychol. 16, 229–250. doi: 10.1123/tsp.16.3.229, PMID: 36626690

[ref24] Guba E. G. Lincoln Y. S. (1994). Competing paradigms in qualitative research. Handbook Qualitative Research. eds. Jowett S. Lavallee D. (Thousand Oaks, CA: Sage Publications, Inc), 105–117.

[ref25] Harwood C. G. Keegan R. J. Smith J. M. J. Raine A. S. (2015). A systematic review of the intrapersonal correlates of motivational climate perceptions in sport and physical activity. Psychol. Sport Exerc. 18, 9–25. doi: 10.1016/j.psychsport.2014.11.005

[ref26] Higgins R. L. Leibowitz R. Q. (1999). “Reality negotiation and coping: the social construction of adaptive outcomes in coping” in The psychology of what works. ed. Snyder C. R. (New York, NY: Oxford University Press), 20–49.

[ref27] Horton P. (2014) in The role of the coach in practical sports coaching. ed. Nash C. (London: Routledge), 31–43.

[ref28] Jekauc D. Vrancic D. Fritsch J. (2024). Insights from elite soccer players: understanding the downward spiral and the complex dynamics of crises. Ger. J. Exerc. Sport Res. 54, 429–441. doi: 10.1007/s12662-024-00968-0

[ref29] Jowett S. (2017). Coaching effectiveness: the coach–athlete relationship at its heart. Curr. Opin. Psychol. 16, 154–158. doi: 10.1016/j.copsyc.2017.05.006, PMID: 28813341

[ref30] Jowett S. Shanmugam V. (2016). “Relational coaching in sport: its psychological underpinnings and practical effectiveness” in Routledge international handbook of sport psychology. eds. Schinke R. J. McGannon K. R. Smith B. (Abingdon: Routledge/Taylor & Francis Group), 471–484.

[ref31] Jowett S. Wachsmuth S. Boardley I. D. Balduck A. L. (2024). The role of emotional intelligence and quality relationships in athletes’ and coaches’ levels of satisfaction: a multi-study analysis. Sports Coach. Rev., 1–24. doi: 10.1080/21640629.2024.2359774

[ref32] Kleinert J. (2003). Erfolgreich aus der sportlichen Krise. Mentales Bewältigen von Formtiefs, Erfolgsdruck, Teamkonflikten und Verletzungen. München: BLV-Verlag.

[ref33] Lara-Bercial S. Mallett C. (2016). The practices and developmental pathways of professional and olympic serial winning coaches. Int. Sport Coach. J. 3, 221–239. doi: 10.1123/iscj.2016-0083

[ref34] Lara-Bercial S. North J. Petrovic L. Minkhorst J. Oltmanns K. Hamalainen K. (2017). The European sport coaching framework. Champaign, IL: Human Kinetics.

[ref35] Lazarus R. S. Folkman S. (1984). Stress, appraisal, and coping. New York, NY: Springer publishing company.

[ref36] Lochbaum M. Stoner E. Hefner T. Cooper S. Lane A. M. Terry P. C. (2022). Sport psychology and performance meta-analyses: a systematic review of the literature. PLoS One 17:e0263408. doi: 10.1371/journal.pone.0263408, PMID: 35171944 PMC8849618

[ref37] Lyle J. (2002). Sports coaching concepts: a framework for coaches’ behaviour. London: Routledge.

[ref38] Mallett C. J. Rynne S. (2015) in Changing role of coaches across development in *Routledge handbook of sport expertise*. eds. Baker J. Farrow D. (London: Routledge), 394–403.

[ref39] Mikuska E. (2017). The importance of piloting or pre-testing semi-structured interviews and narratives. London: Sage Publications, Inc.

[ref40] Nash C. Mallett C. J. (2019) in Effective coaching in football in *football psychology*. eds. Konter E. Beckmann J. Loughead T. M. (London: Routledge), 101–116.

[ref41] Nash C. S. Sproule J. Horton P. (2011). Excellence in coaching: the art and skill of elite practitioners. Res. Q. Exerc. Sport 82, 229–238. doi: 10.1080/02701367.2011.10599750, PMID: 21699102

[ref42] Olusoga P. Butt J. Hays K. Maynard I. (2009). Stress in elite sports coaching: identifying stressors. J. Appl. Sport Psychol. 21, 442–459. doi: 10.1080/10413200903222921

[ref43] Olusoga P. Maynard I. Butt J. Hays K. (2014). Coaching under pressure: mental skills training for sports coaches. Sport Exer. Psychol. Rev. 10, 31–44. doi: 10.53841/bpssepr.2014.10.3.31

[ref44] Olusoga P. Maynard I. Hays K. Butt J. (2012). Coaching under pressure: a study of Olympic coaches. J. Sports Sci. 30, 229–239. doi: 10.1080/02640414.2011.639384, PMID: 22168369

[ref45] Rhind D. J. A. Jowett S. (2010). Relationship maintenance strategies in the coach-athlete relationship: the development of the COMPASS model. J. Appl. Sport Psychol. 22, 106–121. doi: 10.1080/10413200903474472

[ref46] Salovey P. Mayer J. D. (1990). Emotional intelligence. Imagin. Cogn. Pers. 9, 185–211. doi: 10.2190/dugg-p24e-52wk-6cdg, PMID: 22612255

[ref47] Schwandt T. A. (1994). “Constructivist, interpretivist approaches to human inquiry,” in Handbook of Qualitative Research. eds. Denzin N. K. Lincoln Y. S. (Thousand Oaks: Sage Publications Inc), 118–137.

[ref48] Slade K. Jowett S. Rhind D. (2024). Managing challenging situations in the coach–athlete dyad: introducing the grey zone model from the coach perspective. Int. Sport Coach. J., 1–14. [ahead of print]. doi: 10.1123/iscj.2023-0023, PMID: 36626690

[ref49] Smith B. (2018). Generalizability in qualitative research: misunderstandings, opportunities and recommendations for the sport and exercise sciences. Qual. Res. Sport, Exerc. Health 10, 137–149. doi: 10.1080/2159676X.2017.1393221

[ref50] Smith B. McGannon K. R. (2018). Developing rigor in qualitative research: problems and opportunities within sport and exercise psychology. Int. Rev. Sport Exerc. Psychol. 11, 101–121. doi: 10.1080/1750984X.2017.1317357

[ref51] Smith B. Sparkes A. C. (2016) in Interviews: qualitative interviewing in the sport and exercise sciences in Routledge handbook of qualitative research in sport and exercise. eds. Smith B. Sparkes A. C. (London: Routledge), 103–123.

[ref52] Smith B. Sparkes A. Caddick N. (2014) in Judging qualitative research in *research methods in sports coaching*. eds. Nelson L. Groom R. Potrac P. (London: Routledge), 192–201.

[ref53] Sousa H. Clemente F. Gouveia E. Field A. Sarmento H. (2024a). Effects of changing the head coach on soccer team’s performance: a systematic review. Biol. Sport 41, 83–94. doi: 10.5114/biolsport.2024.131816, PMID: 38524815 PMC10955743

[ref54] Sousa H. Sarmento H. Gouveia E. R. Clemente F. M. (2024b). Perception of professional Portuguese soccer players about the replacement of leadership with the season underway - a qualitative study. Int. J. Perform. Anal. Sport, 1–22. doi: 10.1080/24748668.2024.2436802, PMID: 40101104

[ref55] Thelwell R. C. Weston N. J. V. Greenlees I. A. Hutchings N. V. (2008). Stressors in elite sport: a coach perspective. J. Sports Sci. 26, 905–918. doi: 10.1080/02640410801885933, PMID: 18569556

[ref56] Tozetto A. Carvalho H. da Rosa R. Goedert Mendes F. Silva W. Vieira J.. (2019). Coach turnover in top professional brazilian football championship: a multilevel survival analysis. Front. Psychol. 10:1246. doi: 10.3389/fpsyg.2019.01246, PMID: 31244714 PMC6562306

[ref57] Tracy S. J. (2010). Qualitative quality: eight “big-tent” criteria for excellent qualitative research. Qual. Inq. 16, 837–851. doi: 10.1177/1077800410383121

[ref58] Vella S. A. Oades L. G. Crowe T. P. (2010). The application of coach leadership models to coaching practice: current state and future directions. Int. J. Sports Sci. Coach. 5, 425–434. doi: 10.1260/1747-9541.5.3.425, PMID: 39154575

[ref59] Witzel A. Reiter H. (2012). The problem-centred interview: principles and practice. London: SAGE Publications Ltd.

[ref60] Wu Y. L. Shao B. Newman A. Schwarz G. (2021). Crisis leadership: a review and future research agenda. Leadersh. Q. 32:101518. doi: 10.1016/j.leaqua.2021.101518, PMID: 40115879

[ref61] Zhao C. Jowett S. (2022). Coach leadership in a crisis context: investigating effective coach behaviors during the COVID-19 pandemic with a process view. Front. Psychol. 13:1061509. doi: 10.3389/fpsyg.2022.1061509, PMID: 36544439 PMC9760874

