# Supplementary material for: Leading through performance crises: soccer coaches’ insights on their strategies—a qualitative study
Source: Front Psychol. 2025 Apr 2;16:1576717. doi: 10.3389/fpsyg.2025.1576717 (PMC11999996; doi:10.3389/fpsyg.2025.1576717)
Supplement: Supplementary file 2 [file Supplementary_file_2.docx]

Supplementary Material

# SM 2

Table 2. Interview guide 2

| **Content** | **Key question** |
| --- | --- |
| **Part 1: Ice breaker / familiarization** | |
| Familiarization / experience | First, I would like to ask you to tell me about your soccer coaching career. What stages have you gone through? |
| **Part 2: Roles and competencies of the coach** | |
| Definition of the term role / Experience of the coach | Could you define the term “role”?  Could you tell me about the roles you fulfill as a coach and describe them in more detail?  Could you please describe the competencies that you think a coach needs in order to fulfill these roles? |
| Specific situation | Could you describe a specific situation in which these individual roles come into play? |
| **Part 3: Performance crisis and change of roles and competencies** | |
| Definition of a performance crisis | Could you please define a performance crisis? |
| Change of the role during a performance crisis | Could you please describe a specific situation in which you have experienced a crisis yourself and whether and to what extent your role as a coach has changed as a result? |
| Change of the competencies during a performance crisis | Could you please describe how the competencies of a coach change to fulfill the roles in a performance crisis? |
| Intervention strategies in a crisis | With your current experience, how would you deal with such crises today? |
| **Part 4: Closing question** | |
| Open-ended closing question | That’s it from my side. We’ve discussed a few things now. Is there anything from your end that hasn’t been addressed in this interview but you feel is important in this context? Thank you for your time and openness in discussing this topic! |

Note. The interview guide was translated from German to English.
