# Supplementary material for: Leading through performance crises: soccer coaches’ insights on their strategies—a qualitative study
Source: Front Psychol. 2025 Apr 2;16:1576717. doi: 10.3389/fpsyg.2025.1576717 (PMC11999996; doi:10.3389/fpsyg.2025.1576717)
Supplement: Supplementary file 3 [file Supplementary_file_3.docx]

Supplementary Material

# SM 3

Table 3. Implementation of the twenty questions proposed by Braun and Clarke (2020) to assess the quality of thematic analysis (TA) research

| ***Adequate choice and explanation of methods and methodology*** | |
| --- | --- |
| **Question** | **Implementation** |
| 1. Do the authors explain why they are using TA, even if only briefly? | The rationale for employing reflexive thematic analysis (TA) was the following: “Reflexive thematic analysis was chosen as the methodological approach due to our belief that knowledge is inherently co-constructed. In this framework, the researcher acts as an analytic resource, actively and reflexively engaging with the data to generate themes (Braun and Clarke, 2020). Furthermore, reflexive thematic analysis is a suitable tool for identifying patterns in individuals’ behaviors concerning a specific issue. Aligning with the objective of this study, it also aids in producing research for public consumption, such as practice-oriented studies (Braun et al., 2016).” |
| 2. Do the authors clearly specify and justify which type of TA they are using? | We have written the following for the specification in the manuscript: “Specifically, an inductive reflexive thematic analysis (Braun et al., 2016) within a broader deductive framework was employed, using the research question (i.e., What are the roles of a soccer coach during a performance crisis to manage such situations?) to guide the themes.”  We have written the following for the justification in the manuscript: “Reflexive thematic analysis was chosen as the methodological approach due to our belief that knowledge is inherently co-constructed. In this framework, the researcher acts as an analytic resource, actively and reflexively engaging with the data to generate themes (Braun and Clarke, 2020).” |
| 3. Is the use and justification of the specific type of TA consistent with the research questions or aims? | The use and justification of the specific type of TA is consistent with the research question, which we have formulated as follows: “Furthermore, reflexive thematic analysis is a suitable tool for identifying patterns in individuals’ behaviors concerning a specific issue. Aligning with the objective of this study, it also aids in producing research for public consumption, such as practice-oriented studies (Braun et al., 2016).” |
| 4. Is there a good ‘fit’ between the theoretical and conceptual underpinnings of the research and the specific type of TA (i.e., is there conceptual coherence)? | As Braun et al. (2016) noted, “The ‘flexible’ version of TA we have developed offers the researcher robust processes for identifying patterns and interpreting them in various ways, but detaches these from specific, or inbuilt, ontological and epistemological anchors (p. 193)”. This means researchers must make active choices about how to engage with the data: 1) focusing on semantic (explicitly-stated ideas) or latent (implicitly-stated ideas) aspects; 2) using a deductive or inductive approach; and 3) selecting ontological and epistemological frameworks.  In our manuscript, we explained that we assumed an ontological relativism and a constructivist epistemology, coded with a semantic focus, and adopted an inductive orientation within a broader deductive framework. According to Braun et al. (2016), this combination tends to cluster together more ‘naturally’ |
| 5. Is there a good ‘fit’ between the methods of data collection and the specific type of TA? | We elaborated on this question by stating: “Semi-structured interviews were chosen for their methodological coherence, which aligns with our philosophical assumptions and data analysis approach, due to their inherent flexibility.” |
| 6. Is the specified type of TA consistently enacted throughout the paper? | The reviewers are encouraged to answer this question themselves. We employed a coherent combination of ontological relativism, constructivist epistemology, semantic focus, and inductive orientation in our reflexive thematic analysis. Additionally, we did not use other qualitative methods such as data saturation or line-by-line coding. We used the terms ‘generated’ to emphasize that codes and themes did not simply emerge; rather, the researcher acts as an analytical resource, co-constructing knowledge. We aimed to enhance methodological rigor through transparency, addressing a worthy topic, and achieving resonance, rather than relying on a coding frame, multiple independent coders, consensus coding, or inter-rater reliability measures, which are not inherent to reflexive TA. |
| 7. Is there evidence of problematic assumptions about, and practices around, TA? These commonly include:  a) Treating TA as one, homogenous, entity, with one set of – widely agreed on – procedures.  b) Combining philosophically and procedurally incompatible approaches to TA without any acknowledgement or explanation.  c) Confusing summaries of data topics with thematic patterns of shared meaning, underpinned by a core concept.  d) Assuming grounded theory concepts and procedures (e.g., saturation, constant comparative analysis, line-by -line coding) apply to TA without any explanation or justification.  e) Assuming TA is essentialist or realist, or atheoretical.  f) Assuming TA is only a data reduction or descriptive approach and therefore must be supplemented with other methods and procedures to achieve other ends. | a) We are aware that different types of TA exist, each with distinct procedures (for an overview see Braun and Clarke, 2020).  b) See answer to question 6  c) According to Braun and Clarke (2020), themes are conceptualized as “patterns of shared meaning underpinned by a central organizing concept (p. 39)”. For example, the central organizing concept of the role of the Self-Manager is: “Crucially, the role of the Self-Manager ensures coaches avoid being trapped in the downward spirals often apparent during a performance crisis, thereby retaining full access to their mental resources.” A detailed description of the central organizational concept for each theme can be found in the answer to question 15.  d) No grounded theory concepts or procedures were conducted in the present study.  e) As described by Braun et al. (2016), when conducting reflexive TA, researchers must make ‘active’ choices, including conceptual, epistemological, and ontological frameworks. Our ‘active’ choices are detailed in the section ‘philosophical perspectives and design’.  f) As we wrote in the answer to question 3: Reflexive thematic analysis provides a suitable tool for generating patterns in individuals’ practices or behaviors concerning a specific issue and producing research for public consumption, such as practice-oriented research (Braun et al., 2016). Therefore, we believe that reflexive TA does not need to be complemented by other methods or procedures. |
| 8. Are any supplementary procedures or methods justified, and necessary, or could the same results have been achieved simply by using TA more effectively? | No supplementary procedures or methods were carried out. |
| 9. Are the theoretical underpinnings of the use of TA clearly specified (e.g., ontological, epistemological assumptions, guiding theoretical framework(s)), even when using TA inductively (inductive TA does not equate to analysis in a theoretical vacuum)? | We wrote: As researchers “cannot enter a theoretical vacuum when doing TA [thematic analysis]” (Braun and Clarke, 2020, p. 4), it must be acknowledged that pre-existing theoretical assumptions inevitably biased the results. These assumptions included the definition of coaching effectiveness from Côté and Gilbert (2009) as well as literature on excellent coaching (e.g., Bloom et al., 2014; Nash et al., 2011), and performance crisis (Buenemann et al., 2023; Jekauc et al., 2024).”  Furthermore, the ontological and epistemological assumptions can be found in the section ‘Philosophical perspectives and design’ |
| 10. Do the researchers strive to ‘own their perspectives’ (even if only very briefly), their personal and social standpoint and positioning? (This is especially important when the researchers are engaged in social justice-oriented research and when representing the ‘voices’ of marginal and vulnerable groups, and groups to which the researcher does not belong.) | We wrote: “The constructivist framework was well-suited to our research team, which included four practitioners from soccer and two researchers specializing in sport psychology. This diverse composition enabled us to approach the research from both practical and theoretical perspectives, thereby enhancing our understanding and analysis. Together, our experiences and expertise influenced the interpretation and translation of our findings.” |
| 11. Are the analytic procedures used clearly outlined, and described in terms of what the authors actually did, rather than generic procedures? | We followed the recursive six-phase model expressed by Braun and Clarke (2020), noting that our codes were primarily at a semantic level. Additionally, we stated that “In phase 6, during the report writing, inconsistencies, such as vague or overlapping themes, misclassifications, and redundancies became apparent. Consequently, some higher-order themes were merged and redefined, while lower-order themes were reassigned to other higher-order themes to achieve more coherent results.”  For example, the role of the Psychologist was initially divided into two higher-order themes (the Team-Builder and the Psychologist) to clearly demonstrate that some behaviors are more team-oriented while others are more individual-focused. However, after discussions within the research team, we decided to merge these two themes. This decision was based on the understanding that a coach’s behavior cannot be strictly categorized as either team-focused or player-focused. For instance, creating a cohesive unit might seem like a group-level goal, but it requires aligning individual needs and interests with the group. Isolation, as described in Jekauc et al. (2024), occurs at an individual level when players withdraw during a performance crisis, which inevitably affects group dynamics by reducing team cohesion. This ‘dilemma’ led us to converge these two themes.  Additionally, we included a detailed description of the coding process in SM4 – Coding Example. |
| 12. Is there evidence of conceptual and procedural confusion? For example, reflexive TA (e.g., Braun and Clarke 2006) is the claimed approach but different procedures are outlined such as the use of a codebook or coding frame, multiple independent coders and consensus coding, inter-rater reliability measures, and/or themes are conceptualised as analytic inputs rather than outputs and therefore the analysis progresses from theme identification to coding (rather than coding to theme development). | We believe that our responses have demonstrated our deep engagement with the literature on TA. This engagement has enabled us to discern the subtleties and differences between various qualitative approaches. Consequently, we believe we have successfully avoided conceptual and procedural confusion. |
| 13. Do the authors demonstrate full and coherent understanding of their claimed approach to TA? | The reviewers are encouraged to answer this question themselves. |
| A well-developed and justified analysis | |
| 14. Is it clear what and where the themes are in the report? Would the manuscript benefit from some kind of overview of the analysis: listing of themes, narrative overview, table of themes, thematic map? | Braun & Clarke (2016) propose using no more than three theme levels. The headings ‘The fundamental roles’ and ‘The soccer-specific roles’ can serve as overarching themes that structure and organize the manuscript, but they are not analyzed in depth.  To ensure clarity, we named the headings in the results as our higher-order themes (e.g., The Self-Manager, the Soccer Expert, etc.). In these sections, we first stated the central organizing concept for each theme and then listed the associated lower-order themes in italics (e.g., inner balance, self-reflection, and adaptive coping strategies for Self-Manager) to clearly organize the results.  Additionally, we incorporated a figure to provide a comprehensive summary of the analysis. This figure illustrates the higher-order themes along with their corresponding lower-order themes. |
| 15. Are the reported themes topic summaries, rather than ‘fully realised themes’ – patterns of shared meaning underpinned by a central organising concept?  If so, are topic summaries appropriate to the purpose of the research?  ○ If the authors are using reflexive TA, is this modification in the conceptualisation of themes explained and justified?  Have the data collection questions been used as themes?  Would the manuscript benefit from further analysis being undertaken, with the reporting of fully realised themes?  Or, if the authors are claiming to use reflexive TA, would the manuscript benefit from claiming to use a different type of TA (e.g., coding reliability or codebook)? | In order to answer this question, we would like to cite the central organizing concepts of each higher-order theme:  Self-Manager: “Crucially, the role of the Self-Manager ensures coaches avoid being trapped in the downward spirals often apparent during a performance crisis, thereby retaining full access to their mental resources.”  People-Manager: “During periods of performance crises, which are often marked by dynamic and challenging conditions, people-management competencies are necessary in order to understand human behavior, motivate individuals, and create conditions where everyone -including players and board member- feel engaged and valued.”  The Soccer Expert: “The role of the Soccer Expert is characterized by the possession of soccer-specific knowledge and expertise, encapsulated by the principle of being ‘all about the ball’.”  The Psychologist: “The role of the Psychologist emphasizes the importance of understanding and addressing the psychological needs of both individual players as well as the team as a collective.”  The Administrator: “The role of the Administrator is characterized by managing and coordinating the administrative and organizational elements, which are crucial for maintaining team functionality, particularly during performance crises.”  It becomes apparent that each theme addresses different aspects such as intrapersonal competencies, awareness of the player’s needs, and a multidirectional perspective. |
| 16. Is non-thematic contextualising information presented as a theme? (e.g., the first ‘theme’ is a topic summary providing contextualising information, but the rest of the themes reported are fully realised themes). If so, would the manuscript benefit from this being presented as non-thematic contextualising information? | For comprehensibility, we differentiated between the fundamentals (Self-Manager and People-Manager) and the soccer-specific roles (the Soccer Expert, the Psychologist, the Administrator). This differentiation can be seen as a non-thematic contextualising information; however, we have not labeled them as themes in the manuscript. In the ‘General overview of the findings’ section, we wrote: “five higher-order themes (the Self-Manager, the People-Manager, the Soccer Expert, the Psychologist, the Administrator) and their associated lower-order themes were generated.” We hope this demonstrates that the differentiation between the fundamental and the soccer-specific roles should not be seen as a ‘theme’ per se, but rather as a means to enhance readability. |
| 17. In applied research, do the reported themes have the potential to give rise to actionable outcomes? | We believe that performance crises are a highly relevant context for applied research, as they can have wide-ranging effects on all stakeholders involved. The generated themes demonstrate underlying competencies and behaviors on how coaches approach a performance crisis. We believe that these themes have the potential to give rise to actionable outcomes. In addition, we have written an extensive section to ‘Applied implications’ to demonstrate that the results can lead to actionable outcomes. |
| 18. Are there conceptual clashes and confusion in the paper? (e.g. claiming a social constructionist approach while also expressing concern for positivist notions of coding reliability, or claiming a constructionist approach while treating participants’ language as a transparent reflection of their experiences and behaviours) | There should be no conceptual clashes and confusion in this paper. |
| 19. Is there evidence of weak or unconvincing analysis, such as:  ● Too many or two few themes?  ● Too many theme levels?  ● Confusion between codes and themes?  ● Mismatch between data extracts and analytic claims?  ● Too few or too many data extracts?  ● Overlap between themes? | The reviewers are encouraged to answer this question themselves. Since researchers act as an analytical resource, different researchers might produce different results. However, we strived to avoid mismatches between data extracts and analytical claims. Additionally, each theme is built around a central organizing concept, which should reduce overlap between themes. |
| 20. Do authors make problematic statements about the lack of generalisability of their results, and or implicitly conceptualise generalisability as statistical probabilistic generalisability (see Smith 2017)? | No, we have not. We aware that generalizability can be problematic in qualitative research “when it is understood only through one particular type of generalizability, that is, statistical-probabilistic generalizability” (Smith, 2018, p. 139). However, there are different opportunities to generalize in qualitative research. Our approach to this is described in the Trustworthiness section. We aim to generalize by resonance through transferability and naturalistic generalizability. |

# References

Braun, V., and Clarke, V. (2020). One size fits all? What counts as quality practice in (reflexive) thematic analysis? *Qual. Res. Psychol.* 18, 328–352. doi: 10.1080/14780887.2020.1769238

Braun, V., and Clarke, V. (2021). Can I use TA? Should I use TA? Should I not use TA? Comparing reflexive thematic analysis and other pattern-based qualitative analytic approaches. *Couns. Psychother. Res.* 21, 37–47. doi: 10.1002/capr.12360

Braun, V., Clarke, V., & Weate, P. (2016) in Using thematic analysis in sport and exercise research in *Routledge handbook of qualitative research in sport and exercise.* eds. B. Smith, and A. C. Sparkes (London: Routledge), 191-205.

Jekauc, D., Vrancic, D., and Fritsch, J. (2024). Insights from elite soccer players: understanding the downward spiral and the complex dynamics of crises. *Ger. J. Exerc. Sport Res.* 54, 429–441. doi: 10.1007/s12662-024-00968-0

Smith, B. (2018). Generalizability in qualitative research: misunderstandings, opportunities and recommendations for the sport and exercise sciences. *Qualitative Research in Sport, Exercise and Health*, *10*(1), 137-149. https://doi.org/10.1080/2159676X.2017.1393221
