# Supplementary material for: Leading through performance crises: soccer coaches’ insights on their strategies—a qualitative study
Source: Front Psychol. 2025 Apr 2;16:1576717. doi: 10.3389/fpsyg.2025.1576717 (PMC11999996; doi:10.3389/fpsyg.2025.1576717)
Supplement: Supplementary file 4 [file Supplementary_file_4.docx]

Supplementary Material

# SM 4

Please keep in mind that the original interviews were conducted in German. There may be slight variations or subtle nuances that can be found in the German language. Nevertheless, this example is intended to show the coding process in more detail. As described, the coding was carried out on a semantic level, meaning that explicitly expressed meanings and concepts were included. Accordingly, the initial codes were coded close to the text. The excerpt states the following:

I believe that the coach plays an extremely important role, if not the most important one. Because, without putting himself in the spotlight, by the most important role I mean: How does he handle the situation himself, how does he affect the team, does he seem touched, is he shaken, or is he affected but strong. These are different emotional states. A team senses whether a coach believes in them, whether a coach stands behind what he says or if he is just saying slogans in public. For me, it was always about repeatedly saying, ‘Guys, what path are we on, what is our goal, we have already suffered so many setbacks together.’

**Phase 1 = Data familiarization and writing notes**

In this phase, I (the lead author) read the interview transcripts and noted any anomalies or cross-references to other interview transcripts. For example, in this transcript, I repeatedly noticed that the coach refrained from using words such as “me” and “I,” and instead used “we” and “our.” My initial thought was that this conscious or unconscious choice of words could indicate that he is very team-oriented and has internalized this through his choice of words. He does not place himself above the players but sees himself as part of the whole. The last sentence reinforced this impression by stating that they had gone through many setbacks together. He uses this apparent negativity to establish a sense of togetherness. I also drew cross-references to other interviews where I noticed similar patterns.

**The notes:**

Again here: ‘we’, ‘our’. Considers himself part of the whole and does not put himself at the forefront. Nevertheless, he provides guidance and gives strength. Using language as an indicator of internalizing team orientation?

Setbacks suffered: We have already gone through a lot together, a kind of siege mentality, which could imply cohesion.

Authenticity (Coach 6) - Energy (Coach 2) - Leading by example (Coach 2) - Will to win (Coach 2)

**Phase 2 = initial coding**

A total of 7 initial codes were assigned in this excerpt. The codes were as followed:

Table 4. Coding Example

| Code 1 = Centrality of the coach | I believe that the coach plays an extremely important role, if not the most important one |
| --- | --- |
| Code 2 = flat hierarchy, Considering oneself as part of the whole | I believe that the coach plays an extremely important role, if not the most important one. Because, without putting himself in the spotlight |
| Code 3 = lead by example | How does he handle the situation himself, how does he affect the team, does he seem touched, is he shaken, or is he affected but strong. These are different emotional states. A team senses whether a coach believes in them, whether a coach stands behind what he says or if he is just saying slogans in public |
| Code 4 = Authenticity | A team senses whether a coach believes in them, whether a coach stands behind what he says or if he is just saying slogans in public |
| Code 5 = stay focused on the goal | For me, it was always about repeatedly saying, ‘Guys, what path are we on, what is our goal |
| Code 6 & 7 = create unity; all in the same boat | For me, it was always about repeatedly saying, ‘Guys, what path are we on, what is our goal, we have already suffered so many setbacks together.’ |

As you can see, writing the memos influenced the initial generation of codes. Codes such as “flat hierarchy” and “create unity” allude to the notion that the coach sees himself as part of the whole and aims to establish a cohesive team.

**Phase 3: Initial Themes Derived from Initial Codes**

During this phase, the initial codes were categorized into initial themes. The sections corresponding to Codes 2, 6, and 7 emphasize the coach’s perception of himself as an integral part of the team and his efforts to promote a flat hierarchy. Consequently, we identified the theme “Team Builder,” reflecting the coach’s aim to create a cohesive unit. Conversely, the sections related to Codes 3 and 4 focus more on the coach’s external appearance and how he is perceived by others, leading to the identification of the theme “the Leader.”

**Phases 4 & 5: Reviewing Similarities and Differences, and Refining, Defining, and Naming Themes**

After discussing the codes within the research team, the initial theme of “the Leader” was divided into the final themes of “the Self-Manager” and “the People-Manager.” This differentiation was based on the following rationale. Upon re-examining the excerpt:

How does he handle the situation himself, how does he affect the team, does he seem touched, is he shaken, or is he affected but strong. These are different emotional states. A team senses whether a coach believes in them, whether a coach stands behind what he says or if he is just saying slogans in public.

we observed that the theme of “the Leader” was overly generic. The Leader must manage his own emotions and behaviors while also influencing his team to achieve collective goals. The first sentence (“how does he handle the situation himself...”) pertains to his self-assessment and acceptance of the situation, whereas the second sentence (“A team senses whether a coach believes in them...”) clearly addresses his external presentation. In this sense, authenticity can be understood as a communication competence wherein the coach endeavors to motivate his players towards common goals.

In addition, the answer to question 11 explains the reasons for renaming the initial theme “the Team-Builder” to “the Psychologist.”
